# Registry-Based Retrospective Cohort Study of Mortality among Adults Admitted to Intensive Care Units in Istanbul with Hospital Acquired *Pseudomonas aeruginosa* Bloodstream-Infection between 2014–2021

**DOI:** 10.3390/antibiotics13010090

**Published:** 2024-01-17

**Authors:** Okan Derin, Meyha Şahin, Rıdvan Dumlu, Sedef Başgönül, Ahmet Doğukan Bayrak, Şevval Arduç, Sümeyye Bayram, Nurlana Mikaliyova, Arzu Kantürk, Ahsen Öncül, Dilek Yıldız Sevgi, Serap Gençer, Banu Bayraktar, İlyas Dökmetaş, Ali Mert

**Affiliations:** 1 Graduate School of Health Sciences, Epidemiology Doctorate Program, Istanbul Medipol University, 34815 Istanbul, Turkey; 2 Istanbul Şişli Hamidiye Etfal Training and Research Hospital Infectious Diseases and Clinical Microbiology, 34396 Istanbul, Turkey; dogukan9653@gmail.com (A.D.B.); onculahsen@gmail.com (A.Ö.); dileky26@hotmail.com (D.Y.S.); idokmetas@yahoo.com (İ.D.); 3 Faculty of Medicine, Infectious Diseases and Clinical Microbiology, Istanbul Medipol University, 34214 Istanbul, Turkey; meyha.sahin@medipol.edu.tr (M.Ş.); nurlanant@gmail.com (N.M.); 4 Istanbul Prof. Dr. Cemil Taşçıoğlu Training and Research Hospital, Infectious Diseases and Clinical Microbiology, 34384 Istanbul, Turkey; r_dumlu@hotmail.com (R.D.); karabiyiksumeyye@gmail.com (S.B.); drakanturk@gmail.com (A.K.); 5 Department of Infectious Diseases and Clinical Microbiology, Acıbadem Mehmet Ali Aydınlar University, 34752 Istanbul, Turkey; sedefbasgonul@hotmail.com (S.B.); gencerse@gmail.com (S.G.); 6 Hamidiye Faculty of Health Sciences, University of Health Sciences, 34668 Istanbul, Turkey; sevvalaarduc@gmail.com (Ş.A.); bbayraktar43@gmail.com (B.B.); 7 Istanbul Şişli Hamidiye Etfal Training and Research Hospital Microbiology and Clinical Microbiology, 34396 Istanbul, Turkey; 8 Department of Internal Medicine, Istanbul Medipol University, 34815 Istanbul, Turkey

**Keywords:** Pseudomonas, bloodstream infections, intensive-care units

## Abstract

Background: Managing *Pseudomonas aeruginosa* bloodstream infections (BSIs) is challenging due to increasing antimicrobial resistance, limited therapeutic options, and high mortality rates. In this study, we aimed to identify 30-day mortality risk factors and assess infectious diseases consultants’ preferences for combination or monotherapy. Methods: The study was conducted in four hospitals in Istanbul, Turkey, involving 140 adult ICU beds and 336,780 ICU-bed-days between 1 January 2014, and 31 December 2021. A total of 157 patients were included in the study. Cox proportional hazard regression was performed to assess the factors on 30-day mortality. Results: The 30-day mortality rate was 44.6% (70/157). Higher Charlson Comorbidity Index (CCI) score, severe sepsis, primary bloodstream infection, being in COVID-19 pandemic period, and infection caused by MDR strain were associated with higher hazard of 30-day mortality. Combination therapy was more commonly used in patients with BSIs with MDR or DTR (difficult-to-treat) strains but did not significantly improve the hazard of 30-day mortality. Conclusions: Targeted interventions and vigilant management strategies are crucial for patients with defined risk factors. While infectious disease consultants tended to favor combination therapy, particularly for drug-resistant strains, our analysis revealed no significant impact on 30-day mortality hazard. The increased incidence of *P. aeruginosa* BSIs during the pandemic emphasizes the need for infection control measures and appropriate antibiotic prescribing practices.

## 1. Introduction

Managing *Pseudomonas aeruginosa* bloodstream infections (BSIs) remains challenging [1] because of increasing antimicrobial resistance [2,3], limited therapeutic options [4], and high case mortality rates [5]. *P. aeruginosa* is one of the most common pathogens isolated from ventilator-associated pneumonia and BSIs in intensive care units (ICUs) [6].

The clinical relevance of different patterns of antibiotic resistance in invasive *P. aeruginosa* is vital for optimizing patient care, preserving the efficacy of antibiotics, and addressing broader public health concerns related to antimicrobial resistance. According to a combined report of the European Antimicrobial Resistance Surveillance Network Report and the Central Asian and European Surveillance of Antimicrobial Resistance network [7], carbapenem resistance in invasive *P. aeruginosa* isolates shows large differences across European countries. The report concluded that the antimicrobial resistance rates in the WHO Europe region were still high for the microorganisms under surveillance. In Türkiye, carbapenem and multi-drug resistant (MDR), defined as combined resistance to ≥3 antimicrobial groups) *P. aeruginosa* rates were 39% and 28.1%, respectively. The European Committee on Antimicrobial Susceptibility Testing (EUCAST) clinical breakpoints have been widely used in Türkiye since 2014. EUCAST proposed changing the intermediate categories of *P. aeruginosa* in 2015 and 2017 for piperacillin-tazobactam, cefepime, ceftazidime, ceftazidime-avibactam, aztreonam, ciprofloxacin, and levofloxacin (unless screened for other quinolones) [8]. In 2020, the tenth version of EUCAST breakpoint table update was introduced “Susceptible, increased exposure” instead of “Susceptible, standard dosing” for piperacillin, piperacillin/tazobactam, ticarcillin, ticarcillin/clavulanic acid, cefepime, ceftazidime, imipenem, aztreonam, ciprofloxacin, and levofloxacin for *P. aeruginosa* (Supplement S-1) [9]. A guideline for “difficult-to-treat” (DTR) Gram-negative organisms were introduced by the Infectious Diseases Society of America (IDSA) despite disagreements regarding the concept of DTR, which relates to first-line antipseudomonal antibiotics such as beta-lactams and quinolones [10].

Monotherapy or combination therapy may be used in a clinical setting for either an empirical or directed treatment of *P. aeruginosa* BSIs. There is no consensus on whether directed monotherapy or combination therapy is associated with improved outcomes; however, empirical combination antimicrobial prescriptions based on patient risk factors and local resistance data are widely accepted by clinicians [11,12].

The primary endpoint of the study was to assess the impact of monotherapy and combination therapies on 30-day mortality rates. The study’s secondary outcomes of interest were the rates of MDR and DTR based on updated definitions; resistance and mortality of *P. aeruginosa* over time, as well as the association between *P. aeruginosa* mortality at 30 days and resistance patterns.

## 2. Results

**Figure 1:**
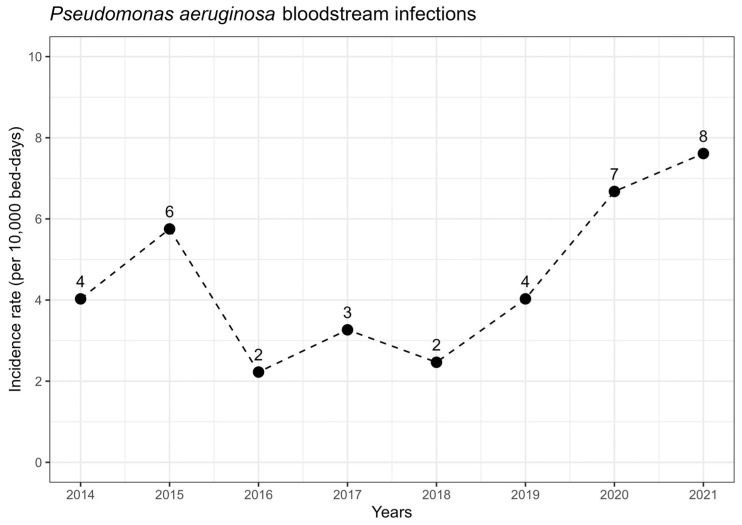
*Pseudomonas aeruginosa* bloodstream infection total incidence (dots) in study centers between 2014–2021. Trend has been presented as dashed lines.

We enrolled 157 patients with *P. aeruginosa* bacteremia in intensive care units. Of these, 100 (64%) were male, with a median age of 68 years (interquartile range, 57–77), and 127 (81%) were mechanically ventilated. Females had a median age of 75 years (IQR, 60–80), significantly older than males with a median age of 64 years (IQR, 56–75, *p* = 0.007). Table 1 summarize the demographic, clinical, and microbiological characteristics of the patients with bloodstream infections caused by *P. aeruginosa* by outcome and therapeutic approaches. The study found a 30-day mortality rate of 44.6% (70/157). The incidence rate per 10,000 bed-days per year is shown in Figure 1.

*P. aeruginosa* BSIs occurred in 84 (54%) and 73 (46%) patients, with incidence rates of 3.58 and 7.17 per 10,000 bed-days during periods 1 and 2, respectively. There were more mechanically ventilated patients in period 2 (n = 66, 90%) than period 1 (n = 61, 73%; *p* = 0.09). There was no significant difference in the source of bacteremia between the two periods, with primary BSIs accounting for 23 (27%) in period 1 and 22 (30%) in period 2. Subclassifications of infection source have been provided in the supplement (Supplement S-5). While recently introduced DTR definition reflected to backwards: the proportion of BSIs caused by DTR *P. aeruginosa* was significantly higher in period 2 (n = 28, 38%) than in period 1 (n = 9, 11%; *p* < 0.001). There was no significant difference in the proportion of BSIs caused by MDR *P. aeruginosa* between periods 1 (n = 22, 26%) and 2 (n = 26, 36%; *p* = 0.2). In period 2, combination therapy approaches were more frequent (*p* = 0.051) (Table 1).

Mortality rates were higher in the inappropriate empirical therapy group (n = 44, 51%) compared with the appropriate empirical therapy group (n = 26 41%; *p* = 0.2). Patients with MDR *P. aeruginosa* BSI were more likely to receive inappropriate empirical therapy compared with those with non-MDR infections (42.0% vs. 15.0%; *p* < 0.001). A total of 21 patients did not receive any empirical antipseudomonal treatment. This group consists of those who either died immediately after the blood culture was drawn (n = 7) or received non-antipseudomonal treatment (n = 14). In summary, the classification is as follows: 57% (n = 86) received inappropriate treatment, while 43% (n = 64) received appropriate empirical antibiotic therapy. Those who received non-appropriate empirical therapy were categorized as such. Antipseudomonal empirical treatment was initiated in 136 patients, with 113 receiving monotherapy and 23 receiving combination therapy. Following the receipt of blood culture reports, the definitive therapy of 87 of 136 (63%) remained the same regime as empirical therapy.

### 2.1. Patient Factors Related to Outcomes and Therapeutic Options

As we presented details on Table 1, we observed higher 30-day mortality among patients with

- a higher median CCI score,
- immunocompromised status,
- individuals with a central line,
- hospitalized within the last three months.

Patients with cerebrovascular events exhibited lower mortality rates. Notably, no patient-specific characteristics were found to be linked to the selection of monotherapy or combination medication.

We performed both univariable and multivariable analyses using Cox proportional hazard analysis (Table 2) to investigate the risk factors associated with 30-day mortality. In the multivariable analysis, we identified several significant factors related to lower 30-day survival probability; these included being in period 2, which corresponds to the COVID-19 pandemic period, having a higher CCI, having a platelet count below 10^5^/L, acute kidney injury, having a primary BSI, and having a BSI caused by a MDR strain. However, we did not find any significant association between combination therapy and 30-day survival. In another analysis, we stratified the model based on mechanical ventilation, and it was observed that being in period 2 still maintains a significant association with mortality. However, it is worth noting that this stratified model exhibited a weaker overall fit. Supplement S-6 depicts multiple stratification and analysis of outcome by period and resistance type.

### 2.2. Source of Bacteremia

Primary BSIs had a higher 30-day mortality (n/N = 24/45 (53 vs. n/N = 4/112 (41%); *p* = 0.054) than secondary BSIs. Table 1 provides specifics on the various secondary BSI subtypes. We further classified subtypes of secondary bloodstream infections, stratified into periods 1 and 2, and detailed the findings in Supplement S-5.

### 2.3. Microbiologic Factors and Resistance Patterns by Years

**Figure 2:**
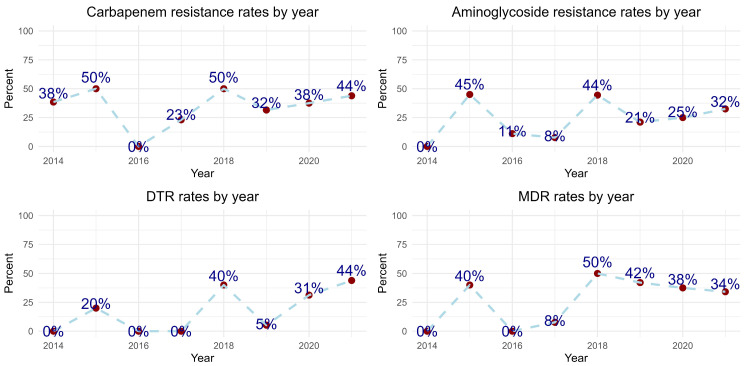
Resistance patterns of *Pseudomonas aeruginosa* isolated bloodstream infections in study centers. Note: Recently defined DTR pattern reflected backwards.

Figure 2 illustrates the trends in aminoglycoside and carbapenem resistance, as well as the DTR and MDR patterns of *P. aeruginosa*. Upon further evaluation of the AST results for imipenem by year, we observed that the “I” (intermediate) result was noted in 5.6%, 6.3%, and 47.5% of cases in 2019, 2020, and 2021, respectively.

Control blood culture results within 7 days were available for a total of 64 patients, with 53 (82%) patients showing blood culture sterilization and 11 (18%) patients not achieving sterilization. We observed that patients who survived had a significantly higher rate of blood culture sterilization within three to seven days of antibiotic therapy (n = 42/53, 79%) compared to those who died (n = 8/11, 73%, *p* = 0.002).

Directed therapy was administered to 148 out of 157 patients. However, nine out of 157 patients (2%) did not receive directed therapy, as they had deceased before culture identification. Among patients with MDR *P. aeruginosa* (n = 48, 31%), combination therapy (n = 30/45, 67%) was more commonly used than monotherapy (n = 15/45, 33%, *p* < 0.001. A total of 81 patients (52%) had BSI with DTR strains. Among them, 33 patients (41%) received monotherapy, while 48 patients (71%) received combination therapy (*p* < 0.001), indicating a higher utilization of combination therapy in the DTR group. The presence of DTR-*P. aeruginosa* or MDR-*P. aeruginosa* was not associated with higher hazard of 30-day mortality in univariate analysis. As stated before, BSI with MDR *P. aeruginosa* was found to have a higher hazard of 30-day mortality in multivariable analysis.

**Figure 3:**
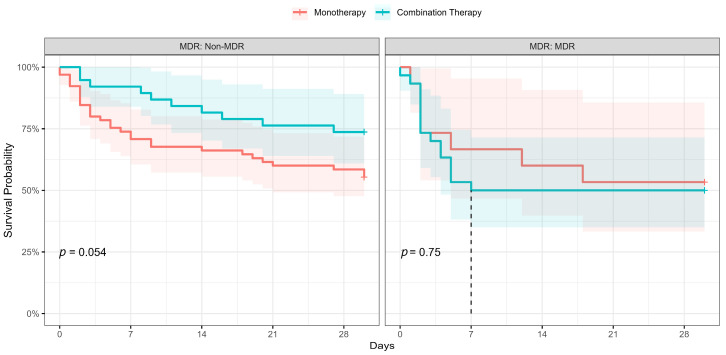
Kaplan–Meier survival analysis monotherapy vs. combination therapy stratified by MDR pattern. The dashed line in the ‘MDR’ group marks the median survival time.

We performed a Kaplan–Meier survival analysis, including a log-rank test, to evaluate the effect of combination therapy on 30-day mortality in both patients with MDR and DTR *P. aeruginosa* bloodstream infections. The results were visualized in Figure 3, which presents a Kaplan–Meier plot. Our analysis revealed no significant association between combination therapy and improved 30-day survival rates among patients with either MDR or DTR *P. aeruginosa* bloodstream infections.

## 3. Discussion

In our study, we showed that combination therapy did not improve the hazard of 30-day mortality.

Monotherapy or combination therapy for *P. aeruginosa* BSIs remain a subject of controversy, and clinicians should base their decision largely on factors such as the patient’s clinical severity, local antimicrobial resistance patterns, and the possibility of the patient being colonized with resistant strains. However, due to a lack of solid evidence, making such a decision can be challenging. In our study, we observed that infectious disease consultants were more inclined towards favoring combination therapy, in patients with DTR or MDR strains BSIs, and during period-2, which corresponded to the COVID-19 pandemic period. This decision-making trend was influenced by factors associated with the colonization of resistant strains such as previous recent antimicrobial exposure. Combination therapy was predominantly selected as carbapenem in conjunction with either an aminoglycoside or polymyxins. Three meta-analyses have examined the use of combination therapy; while two of them discourage its use [13,14], one of the meta-analyses suggests that combination therapy may result in higher survival rates in the case of septic shock [15]. However, these studies have reported an increased incidence of adverse events such as renal toxicity, skin rash, and ototoxicity associated with the use of combination therapy. Our study findings, supported by multivariable analysis (Table 2) and stratified Kaplan–Meier survival analysis (as shown in Figure 3), indicate that the use of combination therapy did not show a significant impact on 30-day mortality in cases of DTR or MDR *P. aeruginosa* bloodstream infections (BSIs) in intensive care units (ICUs). We have not analyzed adverse events. As demonstrated in Table 1, a longer duration of empiric therapy was found to be associated with improved survival. This finding suggests that early diagnosis of BSIs may contribute to better outcomes. Alternatively, it is possible that patients with a more severe clinical condition are more likely to experience early mortality. This would be an issue for further studies. *P. aeruginosa* bacteremia remains a complex challenge. Healthcare workers should customize therapeutic options based on the patient’s characteristics, such as the presence of multiple comorbidities, clinical severity, and factors related to antimicrobial resistance risks like recent hospitalization and antimicrobial consumption. Additionally, consideration of local resistance patterns is essential in making informed treatment decisions. Furthermore, there is a need for the development of novel strategies aimed at preventing and effectively treating *P. aeruginosa* BSIs considering these findings [16,17,18].

A population-based study [19] reported that the median age of individuals with monomicrobial *P. aeruginosa* bloodstream infection was 69 years (with a range of 49–81 years). In our study, the median age of patients with *P. aeruginosa* BSI was 68 years (with a range of 57–77 years). The same study indicated that the incidence of *P. aeruginosa* bacteremia was higher in males than in females, especially after the age of 50 years. Similarly, in our study, 100 (64%) of patients were male (*p* = 0.001), and the median age of females was higher than that of males (75 ± 20 years vs. 64 ± 19 years; *p* = 0.07).

Our study centers utilized standardized international standards for AST methodology, mostly EUCAST, to interpret the antibiotic susceptibilities of *P. aeruginosa*. It is important to note that EUCAST revises clinical breakpoints for antibiotic susceptibilities every year. In 2020, the definition of the Intermediate category was changed to “Susceptible, increased exposure”, and the clinical breakpoint for susceptible was lowered to 0.001 mg/L, which eliminated the “Susceptible, standard exposure”, or formerly Intermediate category for certain antibiotics such as Piperacillin, Piperacillin-tazobactam, Ticarcillin, Ticarcillin-clavulanic acid, Cefepime, Ceftazidime, Aztreonam, Imipenem, Ciprofloxacin, and Levofloxacin. Since the study only included true resistant strains over the study period, possible biases in the data were eliminated. Intermediate resistance rates showed an increasing MIC trend. Surprisingly, the incidence of *P. aeruginosa* BSIs was very low between 2016 and 2018, coupled with lower resistance rates. Our study found the highest rates of resistance to carbapenems, aminoglycosides, DTR, and MDR in 2018. Although these rates decreased slightly in the following years, they are still very high. Furthermore, the isolates mostly belonged to ICU patients, and this trend might reflect the spreading of a selected resistant clone.

The mortality rate in our study (44.5%) is relatively high compared to the literature. Studies have shown that the mortality rate associated with *P. aeruginosa* BSIs ranged from 18% to 58% [20,21]. Furthermore, it has been suggested that changing the resistance profile [22] and inappropriate empirical antibiotic treatment [23] may contribute to the high mortality rates. It is important to note that our study locations have a high prevalence of MDR *P. aeruginosa*, and we also found a higher frequency of inappropriate antimicrobial empirical therapy in the patients with MDR *P. aeruginosa* BSIs. Our results revealed a higher mortality rate in cases of BSIs caused by MDR strains, while no such association was observed for DTR strains, which is consistent with previous findings. This finding suggests that inappropriate empirical therapy may play a significant role in mortality, although we were unable to directly present its impact in our study. Our study was conducted in intensive care units with patients having co-morbidities, and all *P. aeruginosa* bloodstream infections were healthcare-associated. Primary bloodstream infections and secondary bloodstream infections related to pneumonia and hepatobiliary infections, which have been associated with higher mortality rates in the literature [21], accounted for 59% of all *P. aeruginosa* bloodstream infections in our study.

Several factors have been identified in previous studies [24,25,26] to be associated with mortality in *P. aeruginosa* bloodstream infections, including neutropenia, septic shock, high-risk source, inappropriate initial therapy, and high CCI. In our study, univariable analysis revealed that an immunocompromised state, solid cancer, higher CCI, PCT ratio 72 h/0 h > 40%, platelet count < 10^5^ /L, and acute kidney injury were associated with higher mortality, while receiving early initial therapy were protective factors. The higher CCI score, indicating more severe comorbidities, was associated with a higher hazard of 30-day mortality. Immunosuppression is a well-known risk factor for the occurrence of *P. aeruginosa* bloodstream infections (BSIs) and is also associated with its higher mortality rates. Our study’s finding of high mortality rates related to immunosuppression is therefore not unexpected. Additionally, low platelet count (less than 10^5^/L) and acute kidney injury are known to be associated with organ dysfunction caused by sepsis/septic shock, which is a common complication of *P. aeruginosa* BSI. Our study’s findings regarding these factors are consistent with available evidence. Further studies are needed to better understand the complex relationships between these factors and mortality in patients with *P. aeruginosa* BSI.

The COVID-19 pandemic has led to increased hospitalizations and antimicrobial use, contributing to a rise in multi-drug-resistant pathogens and possibly weakening antimicrobial stewardship practices due to healthcare strain [27,28,29]. Two systematic reviews examining the trends of *P. aeruginosa* bloodstream infections during the COVID-19 pandemic period concluded that there was an increased incidence of *P. aeruginosa* BSIs [30,31]. These reviews suggest that factors such as diminished infection control measures, inappropriate antibiotic prescribing, increased incidences of ventilator-associated lower respiratory tract infections, and secondary bacterial infections due to prolonged hospitalization could be possible reasons for the increased incidence of *P. aeruginosa* BSIs. Similarly, our study found an increased incidence of *P. aeruginosa* BSIs during period 2 (as shown in Figure 2). We also observed that period 2 was associated with higher mortality rates (HR: 2.20; 95% CI: 1.17, 4.13). The prevalence of DTR *P. aeruginosa* BSIs was significantly higher in period 2 (n = 28, 76%) compared to period 1 (n = 9, 24%; *p* < 0.001) in our study. This result suggests a potential shift in the epidemiology of *P. aeruginosa* BSIs, indicating an increasing proportion of infections caused by DTR strains. However, in line with existing reviews that emphasize the role of diminished infection control measures, further research is necessary to understand the underlying factors contributing to this change in epidemiology. Furthermore, higher DTR strains of *P. aeruginosa* were identified during period 2, but they were not associated with mortality rates in our study. Ioannou et al proposed a potential link with the increased likelihood of acquiring Pseudomonas infections in ICU settings during the COVID-19 pandemic period. During the study, the implementation of a carbapenem-focused antimicrobial stewardship program led to reduced carbapenem use and potentially influenced the development of antimicrobial resistance, highlighting the effectiveness of such interventions [32].

### 3.1. Strengths of the Study

1. The study included data from four third-level hospitals over an eight-year period in Istanbul, the most populated city in Turkey. This provides a large and diverse dataset.
2. The study focused specifically on *P. aeruginosa* bloodstream infections in intensive care units, which is an important area of research due to the severity of these infections.
3. The study was conducted in a setting with a high prevalence of multidrug-resistant *P. aeruginosa*, which is valuable information for clinicians and researchers.
4. The study used new definitions of resistance, such as DTR and “Susceptible-Increased Dose” and analyzed resistance epidemiology and therapeutic options using these new definitions. This may lead to more effective treatment strategies in the future.
5. Our study innovates by extensively analyzing *Pseudomonas aeruginosa* bloodstream infections in ICUs, focusing on multidrug-resistant strains, and assessing the impact of new resistance definitions and treatment strategies on mortality. Additionally, it explores the epidemiological shifts during the COVID-19 pandemic, providing basic insights into the evolution of antibiotic resistance and infectious diseases in a critical care setting.

### 3.2. Limitations of the Study

1. The study was retrospective, which means that some data may have been missing or incomplete. This could affect the accuracy of these results.
2. Because of the lack of data, some variables could not be included in the multivariable analysis. This may have limited the scope of the study and the conclusions that can be drawn from it.
3. We did not include any variables for assessing clinical severity, such as APACHE-II or the Pitt bacteremia index, due to the unavailability of reliable data from paperwork archives during the study period.

## 4. Materials and Methods

### 4.1. Study Design and Setting

We conducted a registry-based retrospective study involving 140 adult ICU beds and 336,780 ICU-bed-days in four hospitals in Istanbul, Turkey, between 1 January 2014, and 31 December 2021. This study was reported in compliance with the Reporting of studies Conducted using Observational Routinely Collected Data (RECORD) statement for studies conducted using routinely collected health data [33]. Ethical approval was obtained from the Istanbul Medipol University Non-Interventional Clinical Research Ethics Committee, Istanbul, Türkiye (10.06.2022- E-10840098-772.02-3316).

### 4.2. Participants and Definitions

Definitions were explained in Supplement S-2.

**Figure 4:**
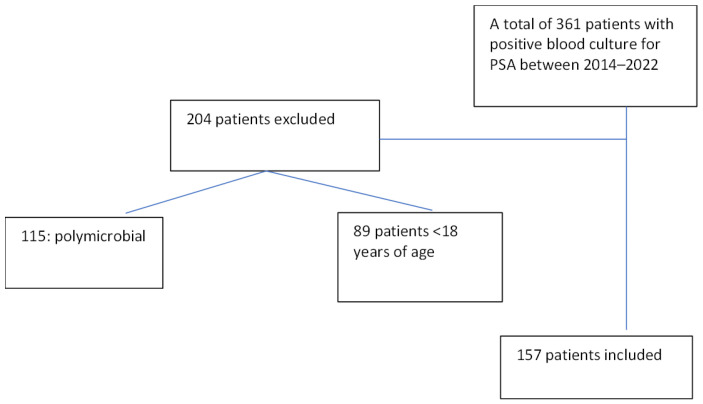
The patient inclusion scheme.

Data were collected from the laboratory information management system for all consecutive ICU patients with positive *P. aeruginosa* blood culture results. Patients aged ≥ 18 years with primary and secondary bloodstream infections 48 h after admission, as defined by the Centers for Disease Control and Prevention (CDC) [34], were included in the study. The polymicrobial results were excluded from the analysis. The final number of participants in the study was 157. The patient inclusion scheme is shown in Figure 4.

Hospital information management systems and archived records were used for the data collection. Demographic and clinical information, previous antibiotic exposure within 3 months, predefined risk factors for *P. aeruginosa* bloodstream infection, and comorbidities were collected, and the Charlson comorbidity index (CCI) was calculated [35]. Secondary BSIs were defined as the apparent microbiological evidence of *P. aeruginosa* foci. It is classified into six categories according to CDC definitions. White blood cell count, neutrophil count, platelet count, C-reactive protein (CRP) level, and procalcitonin level were obtained on the day of positive blood culture. In addition, we collected laboratory data for each parameter based on its half-life (e.g., CRP levels on 72 h). All antimicrobial susceptibility tests (AST) were conducted using either the automated systems (Phoenix^®^ or VITEK^®^) or the Kirby–Bauer disk diffusion method. When necessary, E-test^®^ and broth microdilution (BMD) tests were employed to assess carbapenem or colistin resistance. In the further analysis of carbapenem resistance evaluation, 49 cases were assessed using automated systems, 11 cases with E-test, and 2 cases with BMD (broth microdilution). As for colistin resistance, 54 cases were evaluated with automated systems, 2 cases with E-test, and 81 cases with BMD. The AST reports were provided in accordance with relevant EUCAST clinical breakpoints, except for the second center, which reported AST results using Clinical Laboratories Standards Institute (CLSI) guidelines before 2019, encompassing 34 cases in the study. While DT is a relatively new concept in the clinical setting, MDR has had widely accepted definitions for the past decade. In our study, we opted to utilize a novel definition of MDR *P. aeruginosa* based on the most recent EUCAST clinical breakpoints table. This definition specifically includes combined resistance (only “R” for resistant, excluding “I” for intermediate) to three or more antimicrobial groups, as per the published EUCAST guidelines. This choice was made due to the widespread use of the EUCAST clinical breakpoints table among the four centers involved in the study.

Antimicrobials initiated by the infectious diseases consultant from the best available treatment options were recorded and classified as empiric or directed based on AST results before or after. Further classification was performed as monotherapy or combination therapy. Patient survival or death was recorded within 30 days of a positive blood culture result.

To mitigate the potential impact of missing data on introducing systematic errors, we took several steps, such as implementing an online form to minimize input errors and ensuring the collection of accurate and complete information. Age, sex, comorbidities, study center, and admission date were considered potential confounders and adjusted for using the Cox proportional hazard regression model, as appropriate.

### 4.3. Statistical Analysis

Categorical variables were analyzed using chi-square and Fisher’s exact tests, while continuous variables were analyzed using Student’s *t*-test and Mann–Whitney U test, with a 95% confidence interval. Descriptive statistics were presented as mean ± standard deviation, median [interquartile range], or N (%). ROC analysis was performed to determine the cutoff points of continuous laboratory variables and ratios based on half-life (such as neutrophil count, CRP level, and procalcitonin level), with the cutoff point exhibiting high sensitivity and specificity. A procalcitonin ratio of 72 h to zero h greater than 0.4 was associated with increased mortality (sensitivity = 65.4, specificity = 75.3), and the cutoff for directed therapy was 7.5 days (sensitivity = 77.1%, specificity = 90.8%), based on our findings, and relevant dummy variables were created (Supplement S-3).

Univariable and multivariable analysis using the Cox proportional hazard regression model were performed to assess the risk factors associated with the outcome. We conducted several Cox proportional hazards analysis and constructed the model using a combination of literature review and univariate analysis results. The final model included 11 variables, with period included as potential confounding factor. The model was stratified by the study center. All variables in the final model were selected based on their relevance to the research question and their significant associations in the univariate analysis. The goodness of fit was assessed with concordance index (0.79, se = 0.028), likelihood ratio, Wald, and Score (log rank) tests (*p* < 0.001) supporting the overall significance of the model. Additionally, the Akaike Information Criterion (AIC) value of 381 indicates a favorable balance between model fit and complexity. All variables were included in the analysis after checking for interactions. The rho statistic was used to measure the correlation between the scaled Schoenfeld residuals and time. The *p*-value for the rho statistic was used to test the hypothesis that the correlation is zero. In the final model the covariate “MDR” showed evidence of violation of the proportional hazard assumption (χ^2^ = 5. 4.6594, df = 1, *p* = 0.031) which indicates that the hazard ratio for “MDR” may vary over time. Yet, we opted to keep “MDR” variable because the overall global test for proportional hazards assumption did not reach statistical significance (χ^2^ = 10.8365, df = 11, *p* = 0.457), suggesting that the model’s assumption of proportional hazards is reasonably met for most covariates. The global Schoenfeld test yielded a *p*-value of 0.128, suggesting no significant violation of the proportional hazards assumption (*p* > 0.05). Hence, the relationship between the predictors and hazard rate remains constant over time (output and Schoenfeld’s Residuals plots was given in Supplement S-4). These findings underscore the robustness of our analysis and highlight the predictive value of the identified variables in the context of our study. Right censoring was performed if mortality was not observed within 30 days. We used Kaplan–Meier survival analysis to visualize the time to mortality for combination therapy in overall, MDR and DTR groups and assessed the results with a log-rank test.

## 5. Conclusions

In conclusion, our study provides important insights into the clinical outcomes of *P. aeruginosa* BSIs in ICU patients during the COVID-19 pandemic. The increased incidence of *P. aeruginosa* BSIs and associated mortality in period 2 highlights the potential impact of the pandemic on healthcare systems and patient outcomes. It is crucial to maintain infection control measures and appropriate antibiotic prescribing practices, particularly in the context of the pandemic. Our findings also emphasize the importance of early initiation of appropriate directed therapy for *P. aeruginosa* BSIs in ICUs. Knowledge of local resistance patterns is essential for determining appropriate empiric therapy. Combination therapy was not linked to lower mortality rates. Furthermore, aminoglycosides did not have an impact on mortality rates in the MDR or DTR groups. Given the limited treatment options for MDR and DTR *P. aeruginosa*, there is an urgent need for developing new antimicrobial agents to improve clinical outcomes. Our study highlights the importance of ongoing research in this area to enhance the management and outcomes of *P. aeruginosa* BSIs in critically ill patients.

## Figures and Tables

**Table 1:** Patient Characteristics by Mortality in 30 days and Therapeutic Approach.

	Characteristics by Outcome					Characteristics by Directed Therapy Approach				
Variable	N	Overall, N = 157 ^1^	Survived, N = 87 (55.5%) ^1^	Death, N = 70 (44.5%) ^1^	*p*-Value ^2^	N	Overall, N = 148 ^1^	Monotherapy, N = 80 (54%) ^1^	Combination, N = 68 (46%) ^1^	*p*-Value ^2^
Period	157				0.9	148				0.051
Period 1 (2014–2019)		84 (54%)	47 (56%)	37 (44%)			76 (51%)	47 (59%)	29 (43%)	
Period 2 (2020–2021)		73 (46%)	40 (55%)	33 (45%)			72 (49%)	33 (41%)	39 (57%)	
Patient related factors										
Age	157	68 (57, 77)	65 (56, 76)	68 (58, 78)	0.4	148	66 (56, 77)	68 (56, 76)	65 (56, 77)	0.7
Sex male	157	100 (64%)	55 (55%)	45 (45%)	0.9	148	94 (64%)	50 (62%)	44 (65%)	0.8
CCI	157	4 (2, 6)	4 (2, 5)	5 (3, 7)	0.003	148	4 (2, 6)	4 (2, 6)	4 (2, 6)	>0.9
Mechanical Ventilation	157	127 (81%)	70 (55%)	57 (45%)	>0.9	148	119 (80%)	63 (79%)	56 (82%)	0.7
Immunocompromised	157	29 (18%)	8 (28%)	21 (72%)	<0.001	148	27 (18%)	16 (20%)	11 (16%)	0.5
Cerebrovascular event	154	40 (26%)	32 (80%)	8 (20%)	<0.001	146	39 (27%)	23 (29%)	16 (24%)	0.5
Solid cancer	157	31 (20%)	10 (32%)	21 (68%)	0.004	148	28 (19%)	15 (19%)	13 (19%)	>0.9
Central line	155	91 (59%)	45 (49%)	46 (51%)	0.046	146	82 (56%)	43 (54%)	39 (58%)	0.6
Admission last 3 month	102	59 (58%)	27 (46%)	32 (54%)	0.008	97	55 (57%)	31 (65%)	24 (49%)	0.12
Source of bacteremia ^3^	157				0.054	148				0.4
Primary		45 (29%)	21 (47%)	24 (53%)			43 (29%)	23 (53%)	20 (47%)	
Secondary		112(71%)	66(59%)	46(41%)			105(71%)	57(54%)	48(45%)	
Complicated UTI		13 (8.3%)	8 (61%)	5 (39%)			11 (7.4%)	8 (72%)	3 (28%)	
Pneumonia		39 (25%)	20 (51%)	19 (49%)			34 (23%)	19 (56%)	15 (44%)	
Hepatobiliary		9 (5.7%)	3 (33%)	6 (67%)			9 (6.1%)	7 (78%)	2 (22%)	
Catheter Inf		44 (28%)	28 (64%)	16 (36%)			44 (30%)	20 (46%)	24 (54%)	
Complicated SSTI		7 (4.5%)	7 (100%)	0 (0%)			7 (4.7%)	3 (43%)	4 (57%)	
Microbiologic factors										
TZP Resistance	157	60 (38%)	34 (56%)	26 (44%)	0.6	148	57 (39%)	19 (33%)	38 (67%)	<0.001
Carbapenem Resistance	157	59 (38%)	33 (56%)	26 (44%)	>0.9	148	56 (38%)	22 (39%)	34 (61%)	0.005
Aminoglycoside Resistance	155	40 (26%)	18 (45%)	22 (55%)	0.15	146	39 (27%)	13 (33%)	26 (67%)	0.003
AP Cephalosporin Resistance	154	59 (38%)	34 (58%)	25 (42%)	0.6	145	57 (39%)	20 (35%)	37 (65%)	<0.001
DTR ^3^	157	84 (54%)	43 (52%)	41 (48%)	0.3	148	81 (55%)	33 (41%)	48 (59%)	<0.001
MDR ^3^	157	48 (31%)	23 (48%)	25 (52%)	0.2	148	45 (30%)	15 (33%)	30 (67%)	<0.001
Control Blood Culture (within 3–7 days)	64				0.002	64				0.076
Sterilization achieved		53 (83%)	42 (79%)	11 (21%)			53 (83%)	23 (44%)	30 (56%)	
Not sterilized		11 (17%)	8 (73%)	3 (27%)			11 (17%)	8 (73%)	3 (27%)	
Treatment related factors										
ET duration (days)	157	3.0 (2.0, 6.0)	4.0 (3.0, 7.0)	3.0 (1.0, 4.0)	<0.001	148	3.0 (2.0, 7.0)	3.0 (2.0, 7.0)	3.0 (3.0, 5.2)	0.5
Combination ET	136	23 (17%)	15 (63%)	8 (37%)	0.4	136	23 (17%)	6 (26%)	17 (74%)	0.008
ET with TZP (mono/combi)	145	57 (39%)	34 (60%)	23 (40%)	0.4	141	57 (40%)	38 (66%)	19 (34%)	0.008
ET with CP (mono/combi)	145	69 (48%)	36 (52%)	33 (48%)	0.5	141	69 (49%)	30 (43%)	39 (57%)	0.024
ET with AG (mono/combi)	145	9 (6.2%)	6 (66%)	3 (34%)	0.7	141	9 (6.4%)	1 (11%)	8 (89%)	0.013
ET Polymyxins	145	14 (9.7%)	9 (64%)	5 (46%)	0.5	141	14 (9.9%)	4 (29%)	10 (71%)	0.052
DT duration (days)	157	11 (2, 14)	14 (12, 15)	2 (0, 7)	<0.001	148	12 (3, 14)	10 (3, 14)	14 (5, 14)	0.2
Combination DT	148	68 (46%)	43 (63%)	25 (37%)	0.3					
DT TZP	157	34 (22%)	22 (65%)	12 (35%)	0.2	148	34 (23%)	29 (85%)	5 (15%)	<0.001
DT Carbapenem	157	67 (43%)	43 (73%)	24 (27%)	0.057	148	67 (45%)	22 (33%)	45 (67%)	<0.001
DT Aminoglycoside	157	27 (17%)	22 (81%)	5 (29%)	0.003	148	27 (18%)	3 (11%)	24 (89%)	<0.001
DT Polymyxin	157	34 (22%)	20 (59%)	14 (41%)	0.7	148	34 (23%)	1 (3%)	33 (97%)	<0.001
Bacteremia/sepsis related biochemical factors ^1^										
CRP Ratio (72 h/0 h)	117	0.61 (0.43, 0.76)	0.60 (0.43, 0.75)	0.62 (0.43, 0.94)	0.6	117	0.61 (0.43, 0.76)	0.60 (0.40, 0.75)	0.64 (0.45, 0.82)	0.6
PCT Ratio (72 h/0 h) cutoff ≥ 0.4	103	39 (38%)	22 (29%)	17 (65%)	<0.001	103	39 (38%)	22 (56%)	17 (44%)	0.4
Neutrophil Count Ratio (24 h/0 h)	126	0.75 (0.64, 0.94)	0.74 (0.60, 0.90)	0.77 (0.67, 1.37)	0.053	126	0.75 (0.64, 0.94)	0.77 (0.61, 0.94)	0.74 (0.65, 0.94)	>0.9
Platelet count < 100^3^/L	157	31 (20%)	4 (4.6%)	27 (39%)	<0.001	148	26 (18%)	15 (58%)	11 (42%)	0.7
Acute Kidney Injury	157	43 (27%)	11 (13%)	32 (46%)	<0.001	148	38 (26%)	20 (53%)	18 (47%)	0.8

^1^ Median (IQR) or Frequency (%); ^2^ Wilcoxon rank sum test; Pearson’s Chi-squared test; Fisher’s exact test; ^3^ For definitions and calculations see Supplement S-1. TZP: Piperacillin-Tazobactam, CRP: C-Reactive Protein, PCT: Procalcitonin, ET: Empiric Therapy, DT: Directed Therapy, BC: Blood Culture, UTI: Urinary Tract Infection, SSTI: Skin and Soft Tissue Infection, BSI: Bloodstream Infection.

**Table 2:** Univariable and multivariable analysis by outcome.

	Univariable Cox PH				Multivariable Cox PH			
Characteristic	N (Total)	HR ^1^	95% CI ^1^	*p*-Value	N (Total)	HR ^1^	95% CI ^1^	*p*-Value
Age	157	1.01	1.0, 1.02	0.2	148	1.00	0.98, 1.02	0.8
Gender Male	157	1.06	0.66	1.70	0.8	0.99	0.56, 1.75	>0.9
Period	157							
Period 1	Reference							
Period 2		1.06	0.66, 1.70	0.8	148	2.18	1.14, 4.15	0.018
Immunocompromised state	157	2.70	1.61, 4.51	<0.001				
Solid Cancer	157	2.23	1.34, 3.72	0.002				
CCI	157	1.17	1.08, 1.27	<0.001	148	1.18	1.05, 1.31	0.004
Procalcitonin on blood culture time	149	1.00	1.00, 1.00	0.7				
C reactive protein level on blood culture time	157	1.00	1.00, 1.01	<0.001	148	1.00	1.00, 1.00	0.093
Procalcitonin ratio 72nd h/0 h ≥ 0.4	103	3.90	1.74, 8.77	<0.001				
Platelet count < 10^5^/L	157	4.77	2.91, 7.81	<0.001	148	6.92	3.32, 14.4	<0.001
Acute Kidney Injury	157	3.06	1.91, 4.92	<0.001	148	2.64	1.37, 5.09	0.004
MDR	157	1.51	0.93, 2.47	0.10	148	2.49	1.25, 4.96	0.010
DTR	157	1.42	0.89, 2.29	0.14	148	0.63	0.26, 1.53	0.3
BSI Source	157				148			
Secondary Bloodstream Infections (BSIs)	Reference							
Primary Bloodstream Infection (BSI)		1.39	0.85, 2.27	0.2		2.08	1.12, 3.86	0.020
Blood culture eradication after 3 days	64	0.14	0.06, 0.35	<0.001				
Empirical therapy duration ≥ 2.5 days	157	0.33	0.21, 0.53	<0.001				
Combination therapy	148	0.78	0.47, 1.31	0.4	148	0.62	0.31, 1.22	0.2

^1^ HR = Hazard Ratio, CI = Confidence Interval.

## Data Availability

The datasets generated during and/or analyzed during the current study are not publicly available due to administrative reasons but are available from the corresponding author on reasonable request.

## Supplementary Materials

The following supporting information can be downloaded at: https://www.mdpi.com/article/10.3390/antibiotics13010090/s1, S-1: Definitions of EUCAST breakpoint table update: “Susceptible, increased exposure”. S-2: “Definitions for the Study”, S-3: “ROC analysis of laboratory data and creating dummy variables”. S-4: “Cox Proportional Hazard Model goodness of fit analysis results”, S-5: “Subclassifications of BSI source by period 1 and 2”. S-6 “Multiple Stratification and Analysis of Outcome by Period and Resistance Type”. References [10,36,37,38,39,40,41,42] are cited in the Supplementary Materials.

## Statement of Authors

The study has been presented as an oral presentation at the KLİMİK 2023 congress (SS-091, page 48).

## References

[1] Corona A., De Santis V., Agarossi A., Prete A., Cattaneo D., Tomasini G., Bonetti G., Patroni A., Latronico N. (2023). Antibiotic Therapy Strategies for Treating Gram-Negative Severe Infections in the Critically Ill: A Narrative Review. Antibiotics.

[2] Mancuso G., Midiri A., Gerace E., Biondo C. (2021). Bacterial Antibiotic Resistance: The Most Critical Pathogens. Pathogens.

[3] Al-Orphaly M., Hadi H.A., Eltayeb F.K., Al-Hail H., Samuel B.G., Sultan A.A., Skariah S. (2021). Epidemiology of Multidrug-Resistant *Pseudomonas aeruginosa* in the Middle East and North Africa Region. mSphere.

[4] De Oliveira D.M.P., Forde B.M., Kidd T.J., Harris P.N.A., Schembri M.A., Beatson S.A., Paterson D.L., Walker M.J. (2020). Antimicrobial Resistance in ESKAPE Pathogens. Clin. Microbiol. Rev..

[5] Wang M.G., Liu Z.Y., Liao X.P., Sun R.Y., Li R.B., Liu Y., Fang L.X., Sun J., Liu Y.H., Zhang R.M. (2021). Retrospective Data Insight into the Global Distribution of Carbapenemase-Producing *Pseudomonas aeruginosa*. Antibiotics.

[6] Weiner-Lastinger L.M., Abner S., Edwards J.R., Kallen A.J., Karlsson M., Magill S.S., Pollock D., See I., Soe M.M., Walters M.S. (2020). Antimicrobial-Resistant Pathogens Associated with Adult Healthcare-Associated Infections: Summary of Data Reported to the National Healthcare Safety Network, 2015–2017. Infect. Control Hosp. Epidemiol..

[7] Surveillance of Antimicrobial Resistance in Europe, 2021 Data: Executive Summary. https://www.who.int/europe/publications/i/item/9789289058513.

[8] Kahlmeter G. (2017). EUCAST Proposes to Change the Definition and Usefulness of the Susceptibility Category ‘Intermediate’. Clin. Microbiol. Infect..

[9] Meylan S., Guery B. (2020). In the Name of Common Sense: EUCAST Breakpoints and Potential Pitfalls. Clin. Microbiol. Infect..

[10] Tamma P.D., Aitken S.L., Bonomo R.A., Mathers A.J., van Duin D., Clancy C.J. (2021). Infectious Diseases Society of America Guidance on the Treatment of Extended-Spectrum β-Lactamase Producing Enterobacterales (ESBL-E), Carbapenem-Resistant Enterobacterales (CRE), and *Pseudomonas aeruginosa* with Difficult-to-Treat Resistance (DTR-*P. Aeruginosa*). Clin. Infect. Dis..

[11] Peña C., Suarez C., Ocampo-Sosa A., Murillas J., Almirante B., Pomar V., Aguilar M., Granados A., Calbo E., Rodríguez-Baño J. (2013). Effect of Adequate Single-Drug vs Combination Antimicrobial Therapy on Mortality in *Pseudomonas aeruginosa* Bloodstream Infections: A Post Hoc Analysis of a Prospective Cohort. Clin. Infect. Dis..

[12] Foster R.A., Troficanto C., Bookstaver P.B., Kohn J., Justo J.A., Al-Hasan M.N. (2019). Utility of Combination Antimicrobial Therapy in Adults with Bloodstream Infections Due to Enterobacteriaceae and Non-Fermenting Gram-Negative Bacilli Based on In Vitro Analysis at Two Community Hospitals. Antibiotics.

[13] Safdar N., Handelsman J., Maki D.G. (2004). Does Combination Antimicrobial Therapy Reduce Mortality in Gram-Negative Bacteraemia? A Meta-Analysis. Lancet Infect. Dis..

[14] Paul M., Benuri-Silbiger I., Soares-Weiser K., Leibovici L. (2004). β Lactam Monotherapy versus β Lactam-Aminoglycoside Combination Therapy for Sepsis in Immunocompetent Patients: Systematic Review and Meta-Analysis of Randomised Trials. BMJ.

[15] Afonso E., Conoscenti E., Blot S. (2020). Combination Antimicrobial Therapy in *Pseudomonas aeruginosa* Bacteremia. Eur. J. Pediatr..

[16] Miethke M., Pieroni M., Weber T., Brönstrup M., Hammann P., Halby L., Arimondo P.B., Glaser P., Aigle B., Bode H.B. (2021). Towards the Sustainable Discovery and Development of New Antibiotics. Nat. Rev. Chem..

[17] Losito A.R., Raffaelli F., Del Giacomo P., Tumbarello M. (2022). New Drugs for the Treatment of *Pseudomonas aeruginosa* Infections with Limited Treatment Options: A Narrative Review. Antibiotics.

[18] Chaïbi K., Jaureguy F., Do Rego H., Ruiz P., Mory C., El Helali N., Mrabet S., Mizrahi A., Zahar J.R., Pilmis B. (2023). What to Do with the New Antibiotics?. Antibiotics.

[19] Al-Hasan M.N., Wilson J.W., Lahr B.D., Eckel-Passow J.E., Baddour L.M. (2008). Incidence of *Pseudomonas aeruginosa* Bacteremia: A Population-Based Study. Am. J. Med..

[20] Tam V.H., Rogers C.A., Chang K.T., Weston J.S., Caeiro J.P., Garey K.W. (2010). Impact of Multidrug-Resistant *Pseudomonas aeruginosa* Bacteremia on Patient Outcomes. Antimicrob. Agents Chemother..

[21] Vitkauskiene A., Skrodeniene E., Dambrauskiene A., Macas A., Sakalauskas R. (2010). *Pseudomonas aeruginosa* Bacteremia: Resistance to Antibiotics, Risk Factors, and Patient Mortality. Medicina.

[22] Zhang Y., Li Y., Zeng J., Chang Y., Han S., Zhao J., Fan Y., Xiong Z., Zou X., Wang C. (2020). Risk Factors for Mortality of Inpatients with *Pseudomonas aeruginosa* Bacteremia in China: Impact of Resistance Profile in the Mortality. Infect. Drug Resist..

[23] Park S.Y., Park H.J., Moon S.M., Park K.H., Chong Y.P., Kim M.N., Kim S.H., Lee S.O., Kim Y.S., Woo J.H. (2012). Impact of Adequate Empirical Combination Therapy on Mortality from Bacteremic *Pseudomonas aeruginosa* Pneumonia. BMC Infect. Dis..

[24] Cheong H.S., Kang C.I., Wi Y.M., Kim E.S., Lee J.S., Ko K.S., Chung D.R., Lee N.Y., Song J.H., Peck K.R. (2008). Clinical Significance and Predictors of Community-Onset *Pseudomonas aeruginosa* Bacteremia. Am. J. Med..

[25] Kang C.I., Kim S.H., Kim H.B., Park S.W., Choe Y.J., Oh M.D., Kim E.C., Choe K.W. (2003). *Pseudomonas aeruginosa* Bacteremia: Risk Factors for Mortality and Influence of Delayed Receipt of Effective Antimicrobial Therapy on Clinical Outcome. Clin. Infect. Dis..

[26] Zhang Y., Chen X.L., Huang A.W., Liu S.L., Liu W.J., Zhang N., Lu X.Z. (2016). Mortality Attributable to Carbapenem-Resistant *Pseudomonas aeruginosa* Bacteremia: A Meta-Analysis of Cohort Studies. Emerg. Microbes Infect..

[27] Shbaklo N., Lupia T., De Rosa F.G., Corcione S. (2021). Infection Control in the Era of COVID-19: A Narrative Review. Antibiotics.

[28] Hawkins B.K., Walker S.D., Shorman M.A. (2023). Missed Opportunities for Antifungal Stewardship during the COVID-19 Era. Antibiotics.

[29] Malik S.S., Mundra S. (2022). Increasing Consumption of Antibiotics during the COVID-19 Pandemic: Implications for Patient Health and Emerging Anti-Microbial Resistance. Antibiotics.

[30] Ng Q.X., Ong N.Y., Lee D.Y.X., Yau C.E., Lim Y.L., Kwa A.L.H., Tan B.H. (2023). Trends in *Pseudomonas aeruginosa* (*P. Aeruginosa*) Bacteremia during the COVID-19 Pandemic: A Systematic Review. Antibiotics.

[31] Sloot R., Nsonwu O., Chudasama D., Rooney G., Pearson C., Choi H., Mason E., Springer A., Gerver S., Brown C. (2022). Rising Rates of *Hospital-Onset Klebsiella* spp. and *Pseudomonas aeruginosa* Bacteraemia in NHS Acute Trusts in England: A Review of National Surveillance Data, August 2020–February 2021. J. Hosp. Infect..

[32] Ioannou P., Alexakis K., Maraki S., Kofteridis D.P. (2023). Pseudomonas Bacteremia in a Tertiary Hospital and Factors Associated with Mortality. Antibiotics.

[33] Von Elm E., Altman D.G., Egger M., Pocock S.J., Gøtzsche P.C., Vandenbroucke J.P. (2007). The Strengthening the Reporting of Observational Studies in Epidemiology (STROBE) Statement: Guidelines for Reporting Observational Studies. Epidemiology.

[34] Centers for Disease Control and Prevention; NCZID; DHQP Bloodstream Infection Event (Central Line-Associated Bloodstream Infection and Non-Central Line Associated Bloodstream Infection) Device Assoc. Modul. BSI 2022, 1–48. https://www.cdc.gov/nhsn/psc/bsi/index.html.

[35] Charlson M.E., Pompei P., Ales K.L., MacKenzie C.R. (1987). A New Method of Classifying Prognostic Comorbidity in Longitudinal Studies: Development and Validation. J. Chronic Dis..

[36] Hall K.K., Lyman J.A. (2006). Updated Review of Blood Culture Contamination. Clin. Microbiol. Rev..

[37] Freifeld A.G., Bow E.J., Sepkowitz K.A., Boeckh M.J., Ito J.I., Mullen C.A., Raad I.I., Rolston K.V., Young J.-A.H., Wingard J.R. (2011). Clinical Practice Guideline for the Use of Antimicrobial Agents in Neutropenic Patients with Cancer: 2010 Update by the Infectious Diseases Society of America. Clin. Infect. Dis..

[38] Kellum J.A., Lameire N., Aspelin P., Barsoum R.S., Burdmann E.A., Goldstein S.L., Herzog C.A., Joannidis M., Kribben A., Levey A.S. (2012). Improving global outcomes (KDIGO) acute kidney injury work group. KDIGO clinical practice guideline for acute kidney injury. Kidney Int. Suppl..

[39] The European Committee on Antimicrobial Susceptibility Testing Breakpoint Tables for Interpretation of MICs and Zone Diameters, Version 10.0, 2020, 1–77. http://www.eucast.org/clinical_breakpoints.

[40] Clinical & Laboratory Standards Institute: CLSI Guidelines n.d. https://clsi.org/.

[41] Kadri S.S., Adjemian J., Lai Y.L., Spaulding A.B., Ricotta E., Prevots D.R., Palmore T.N., Rhee C., Klompas M., Dekker J.P. (2018). Difficult-to-Treat Resistance in Gram-negative Bacteremia at 173 US Hospitals: Retrospective Cohort Analysis of Prevalence, Predictors, and Outcome of Resistance to All First-line Agents. Clin. Infect. Dis..

[42] Rossolini G.M., Bochenska M., Fumagalli L., Dowzicky M. (2021). Trends of major antimicrobial resistance phenotypes in enterobacterales and gram-negative non-fermenters from ATLAS and EARS-net surveillance systems: Italian vs. European and global data, 2008–2018. Diagn. Microbiol. Infect. Dis..

