# Supplementary material for: Registry-Based Retrospective Cohort Study of Mortality among Adults Admitted to Intensive Care Units in Istanbul with Hospital Acquired Pseudomonas aeruginosa Bloodstream-Infection between 2014–2021
Source: Antibiotics (Basel). 2024 Jan 17;13(1):90. doi: 10.3390/antibiotics13010090 (PMC10812563; doi:10.3390/antibiotics13010090)
Supplement: Supplementary file 1 [file antibiotics-13-00090-s001.zip › antibiotics-2781793.supplementary.pdf]

Supplementary Materials

S-1: Definitions of EUCAST breakpoint table update: “Susceptible, increased exposure”

"6. EUCAST breakpoints are used to categorize results into three susceptibility categories:

S - **Susceptible, standard dosing regimen**: A microorganism is categorized as Susceptible, standard dosing regimen, when there is a high likelihood of therapeutic success using a standard dosing regimen of the agent.

I - **Susceptible, increased exposure**: A microorganism is categorized as Susceptible, increased exposure* when there is a high likelihood of therapeutic success because exposure to the agent is increased by adjusting the dosing regimen or by its concentration at the site of infection.

R - **Resistant**: A microorganism is categorized as Resistant when there is a high likelihood of therapeutic failure even when there is increased exposure.

*Exposure is a function of how the mode of administration, dose, dosing interval, infusion time, as well as distribution and excretion of the antimicrobial agent will influence the infecting organism at the site of infection."

https://www.eucast.org/clinical_breakpoints

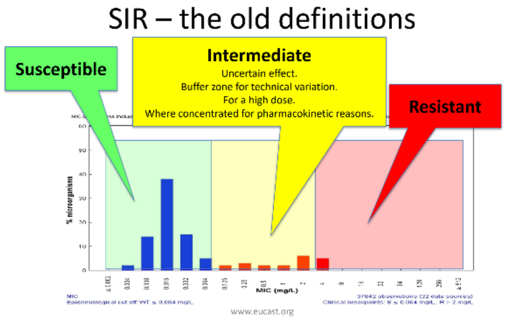

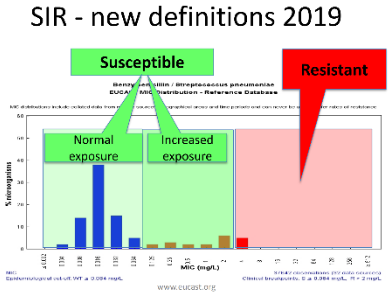

S-2: Definitions for the Study Sample

*Table 1. Definitions**

Bloodstream infection[1]	A laboratory-confirmed positive blood culture with PSA that may be the result of an infection at another body site (secondary BSI) or not (primary BSI)
Polymicrobial blood culture	Detection of one or more microorganisms other than PSA in the same sample
Neutropenia	A granulocyte count < 500 cells/µL or < 1000 cells/µL with an expected decline greater than or equal to 500 cells/µL over the next 48 hours[2]
Acute Kidney Injury	Increase in serum creatinine by ≥0.3 mg/dL (≥26.5 micromole/L) within 48 hours, or<br><br>Increase in serum creatinine to ≥1.5 times baseline, which is known or presumed to have occurred within the prior seven days,<br><br>or<br><br>Urine volume <0.5 mL/kg/hour for six hours[3]

Antimicrobial breakpoints[4,5]	CLSI or EUCAST guidelines applicable to the year of admission were utilized.
Carbapenem-resistant	Any isolate that tested (R) to at least 1 of these: imipenem,<br>meropenem
Aminoglycoside-resistant	Any isolate that tested (R) to at least 1 of these: amikacin,<br>gentamicin, tobramycin
Difficult-to-Treat[6,7]	Intermediate or resistant to all reported agents in carbapenem, β-lactam, and fluoroquinolone categories (including additional agents when results available)
Multidrug Resistant[8]	Any isolate that tested (R) to at least 1 drug in at least<br>3 of these categories:<br>1. Extended spectrum cephalosporins (ceftazidime, cefepime)<br>2. Fluoroquinolones (ciprofloxacin, levofloxacin)<br>3. Aminoglycosides (amikacin, gentamicin, tobramycin)<br>4. Carbapenems (imipenem, meropenem, doripenem)<br>5. Piperacillin Group (piperacillin, piperacillin/tazobactam)
Empiric therapy	Antibiotic treatment administered in the event of a suspected infection prior to the availability of microbiology results
Directed therapy	Antibiotic therapy administered for BSI in accordance with available microbiology results
Periods	Period 1: 2014-2019<br>Period 2: 2020-2021

Definitions was modified according to EUCAST definitions after 2019

[1] Hall, K.K.; Lyman, J.A. Updated Review of Blood Culture Contamination. Clin. Microbiol. Rev. **2006**, *19*, 788–802, https://doi.org/10.1128/cmr.00062-05

[2] Freifeld, A.G.; Bow, E.J.; Sepkowitz, K.A.; Boeckh, M.J.; Ito, J.I.; Mullen, C.A.; Raad, I.I.; Rolston,
K.V.; Young, J.-A.H.; Wingard, J.R. Clinical Practice Guideline for the Use of Antimicrobial Agents in Neutropenic Patients with Cancer: 2010 Update by the Infectious Diseases Society of America. Clin. Infect. Dis. **2011**, *52*, e56–e93, https://doi.org/10.1093/cid/cir073

[3] Kellum, J.A.; Lameire, N.; Aspelin, P.; Barsoum, R.S.; Burdmann, E.A.; Goldstein, S.L.; Herzog, C.A.; Joannidis, M.; Kribben, A.; Levey, A.S.; et al. Improving global outcomes (KDIGO) acute kidney injury work group. KDIGO clinical practice guideline for acute kidney injury. Kidney Int. Suppl. **2012**, *2*, 1–138, doi:https://doi.org/10.1038/kisup.2012.1.

[4] The European Committee on Antimicrobial Susceptibility Testing. Breakpoint Tables for Interpretation of MICs and Zone Diameters, Version 10.0, 2020, 0–77. Available online: http://www.eucast.org/clinical_breakpoints (accessed January 20, 2023).

[5] Clinical & Laboratory Standards Institute: CLSI Guidelines n.d. https://clsi.org/ (accessed January 20, 2023).

[6] Tamma, P.D.; Aitken, S.L.; Bonomo, R.A.; Mathers, A.J.; van Duin, D.; Clancy, C.J. Infectious Diseases Society of America Guidance on the Treatment of Extended-Spectrum β-Lactamase Producing Enterobacterales (ESBL-E), Carbapenem-Resistant Enterobacterales (CRE), and Pseudomonas Aeruginosa with Difficult-to-Treat Resistance (DTR-P. Aeruginosa). Clin. Infect. Dis. 2021, 72, e169–e183. https://doi.org/10.1093/CID/CIAA1478.

[7] Kadri, S.S.; Adjemian, J.; Lai, Y.L.; Spaulding, A.B.; Ricotta, E.; Prevots, D.R.; Palmore, T.N.; Rhee, C.; Klompas, M.; Dekker, J.P.; et al. Difficult-to-Treat Resistance in Gram-negative Bacteremia at 173 US Hospitals: Retrospective Cohort Analysis of Preva-lence, Predictors, and

Outcome of Resistance to All First-line Agents. Clin. Infect. Dis. 2018, 67, 1803–1814, doi:10.1093/cid/ciy378.
[8] Rossolini, G.M.; Bochenska, M.; Fumagalli, L.; Dowzicky, M. Trends of major antimicrobial resistance phenotypes in entero-bacterales and gram-negative non-fermenters from ATLAS and EARS-net surveillance systems: Italian vs. European and global data, 2008-2018. Diagn. Microbiol. Infect. Dis. 2021, 101, 115512, https://doi.org/10.1016/j.diagmicrobio.2021.115512.

S-3: ROC analysis of laboratory data and creating dummy variables.

Finding cutoff values for crp_72_0, pct_72_0 and neut_24_0

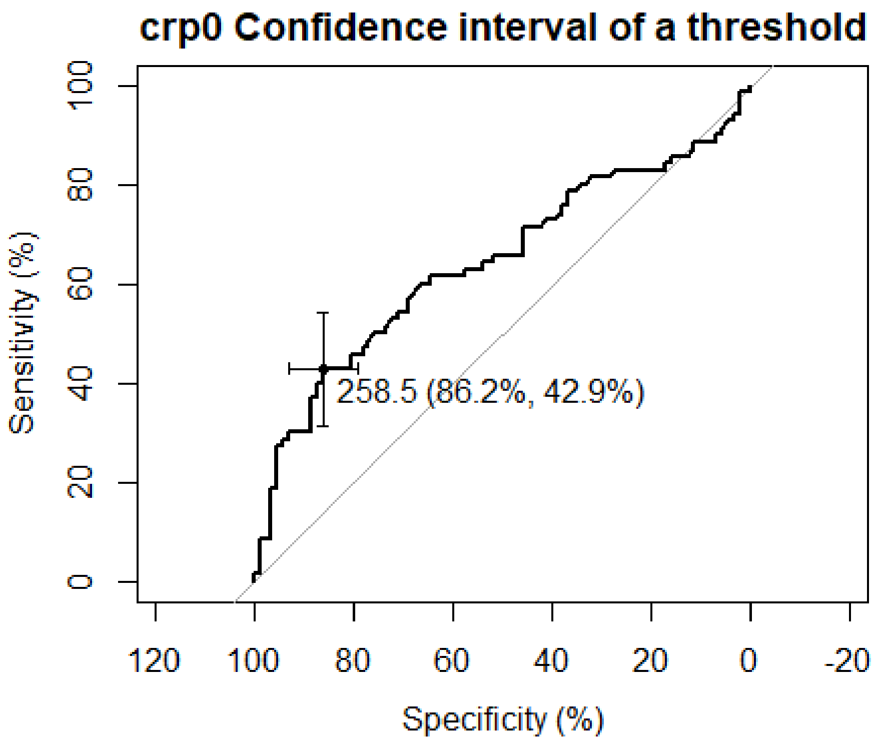

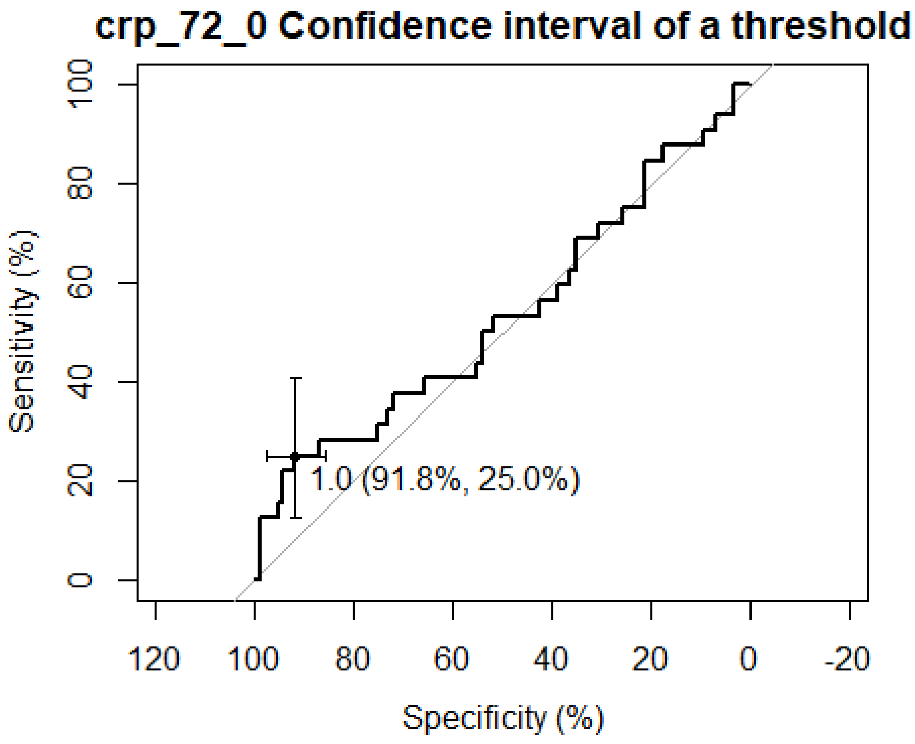

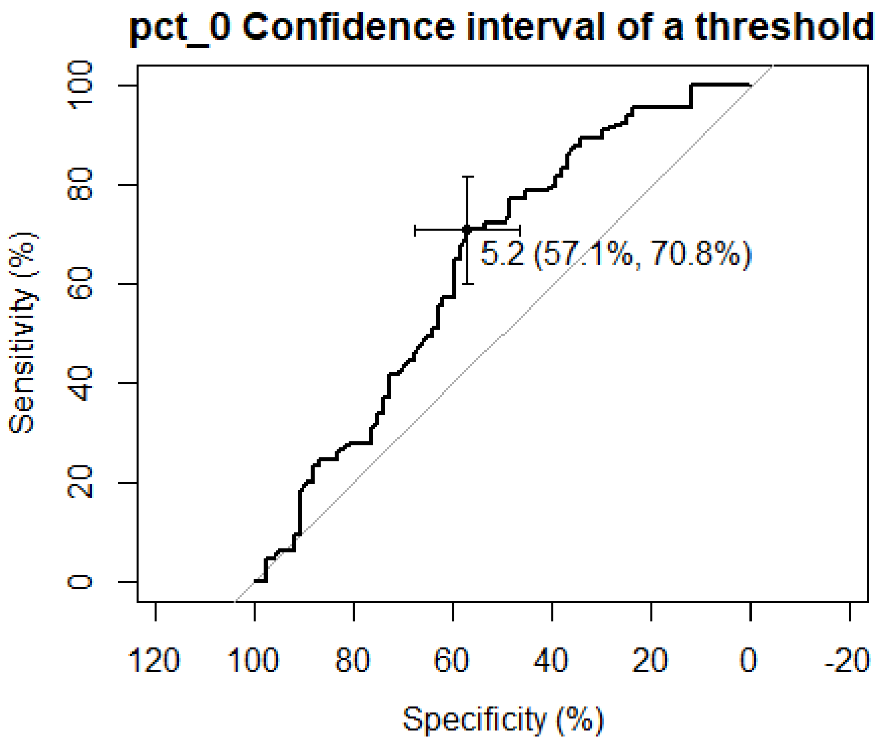
pct_0 Confidence interval of a threshold
5.2 (57.1%, 70.8%)
Sensitivity (%)
Specificity (%)
0
20
40
60
80
100
120
100
80
60
40
20
0
-20

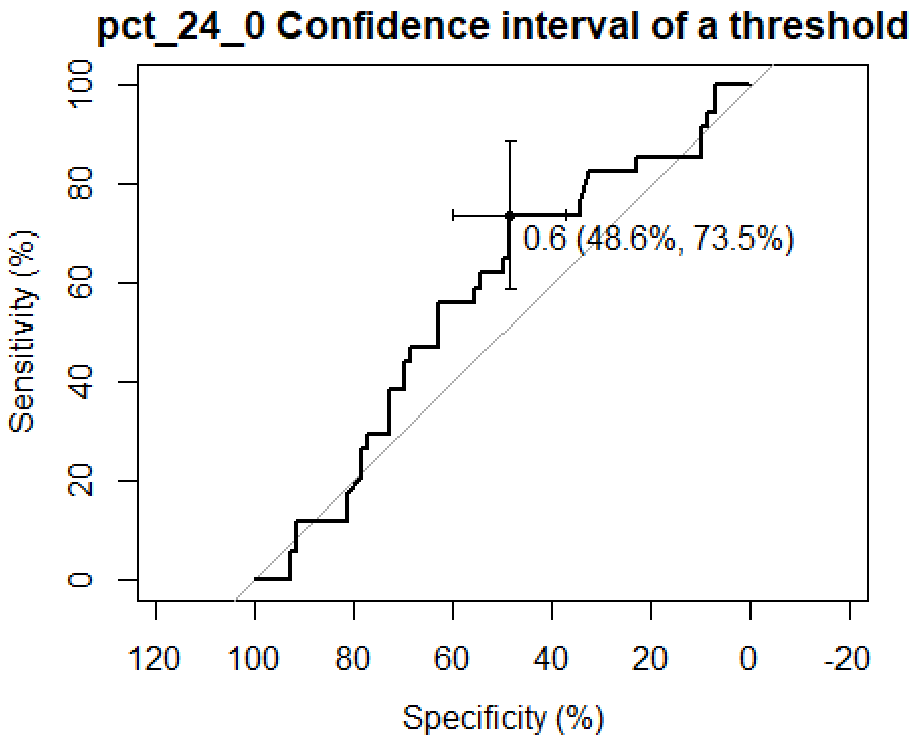
pct_24_0 Confidence interval of a threshold
0.6 (48.6%, 73.5%)
Sensitivity (%)
Specificity (%)
0
20
40
60
80
100
120
100
80
60
40
20
0
-20

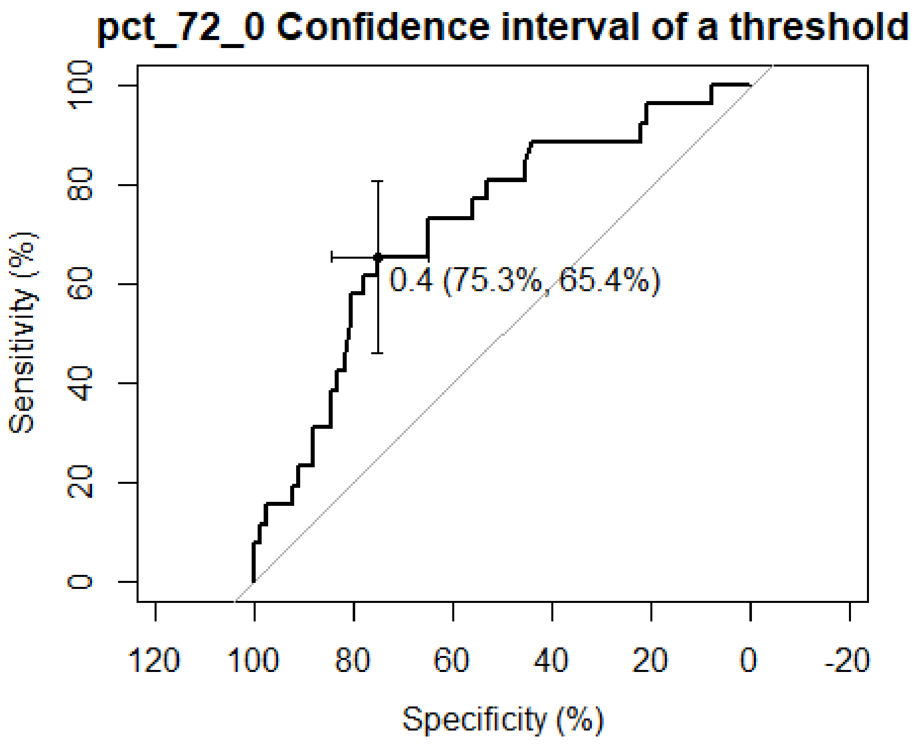
pct_72_0 Confidence interval of a threshold
Sensitivity (%)
Specificity (%)
0.4 (75.3%, 65.4%)
0
20
40
60
80
100
120
100
80
60
40
20
0
-20

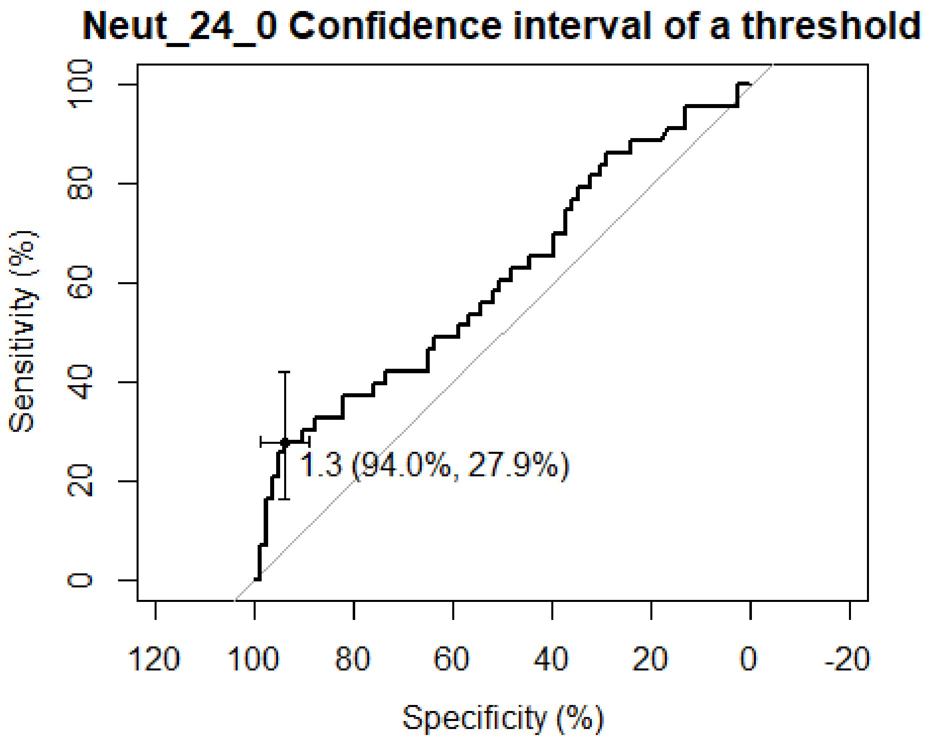
Neut_24_0 Confidence interval of a threshold
Sensitivity (%)
Specificity (%)
1.3 (94.0%, 27.9%)
0
20
40
60
80
100
120
100
80
60
40
20
0
-20

S-4: Cox Proportional Hazard Model goodness of fit analysis results

n= 148, number of events= 61 , ## (9 observations deleted due to missingness)

##

## E-Table 1: Cox Model Proportional Hazards Model

	exp(coef)	exp(-coef)	lower .95	upper .95
mono_combiCombination	0.6168	1.6212	0.3114	1.222
age	0.9979	1.0021	0.9776	1.019
cci	1.1758	0.8505	1.0542	1.311
genderMale	0.9891	1.0111	0.5599	1.747
PeriodPeriod 2	2.1750	0.4598	1.1407	4.147
DTRDTR	0.6280	1.5923	0.2583	1.527
MDRMDR	2.4893	0.4017	1.2494	4.960
sourcePimary	2.0828	0.4801	1.1245	3.858
crp_0	1.0020	0.9980	0.9997	1.004
plt_lower_100Yes	6.9167	0.1446	3.3199	14.411
akiYes	2.6393	0.3789	1.3680	5.092

```
Concordance= 0.792  (se = 0.028 )
Likelihood ratio test= 66.01  on 11 df,   p=0.0000000007
Wald test            = 53.82  on 11 df,   p=0.0000001
Score (logrank) test = 70.67  on 11 df,   p=0.00000000009
AIC= 381
```

## E-Table 3: Cox Model Proportional Hazards Assumption Test

	chisq	df	p
mono_combi	0.3659	1	0.545
age	0.5397	1	0.463
cci	0.0511	1	0.821
gender	0.0785	1	0.779
Period	0.6620	1	0.416
DTR	1.9585	1	0.162
MDR	4.6594	1	0.031
source	0.5369	1	0.464
crp_0	0.0196	1	0.889

```
plt_lower_100  2.2264  1 0.136
aki            2.5342  1 0.111
GLOBAL        10.8365 11 0.457
```

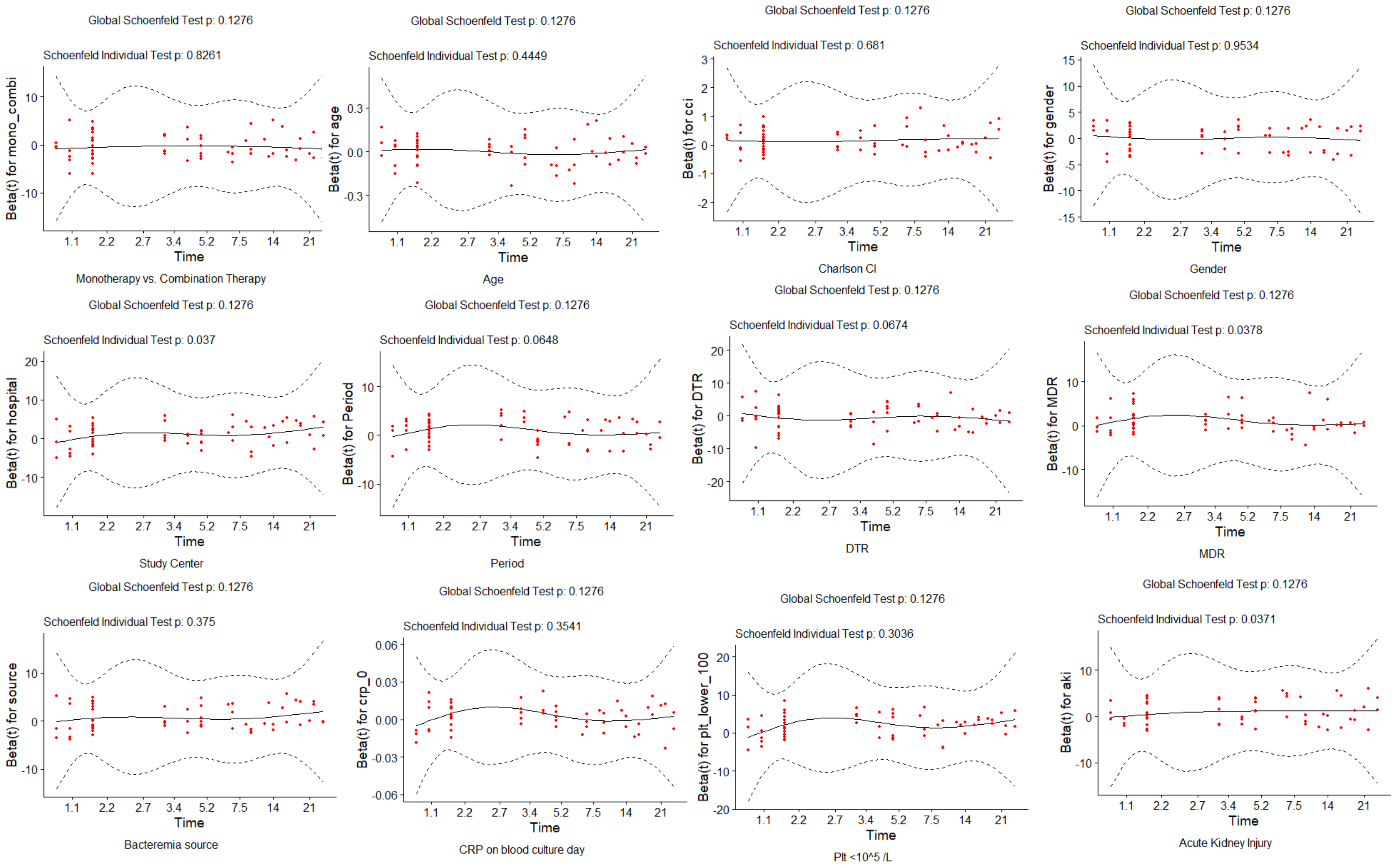
Global Schoenfeld Test p: 0.1276
Schoenfeld Individual Test p: 0.8261
Beta(t) for mono_combi
Time
Monotherapy vs. Combination Therapy
Global Schoenfeld Test p: 0.1276
Schoenfeld Individual Test p: 0.4449
Beta(t) for age
Time
Age
Global Schoenfeld Test p: 0.1276
Schoenfeld Individual Test p: 0.681
Beta(t) for cci
Time
Charlson CI
Global Schoenfeld Test p: 0.1276
Schoenfeld Individual Test p: 0.9534
Beta(t) for gender
Time
Gender
Global Schoenfeld Test p: 0.1276
Schoenfeld Individual Test p: 0.037
Beta(t) for hospital
Time
Study Center
Global Schoenfeld Test p: 0.1276
Schoenfeld Individual Test p: 0.0648
Beta(t) for Period
Time
Period
Global Schoenfeld Test p: 0.1276
Schoenfeld Individual Test p: 0.0674
Beta(t) for DTR
Time
DTR
Global Schoenfeld Test p: 0.1276
Schoenfeld Individual Test p: 0.0378
Beta(t) for MDR
Time
MDR
Global Schoenfeld Test p: 0.1276
Schoenfeld Individual Test p: 0.375
Beta(t) for source
Time
Bacteremia source
Global Schoenfeld Test p: 0.1276
Schoenfeld Individual Test p: 0.3541
Beta(t) for crp_0
Time
CRP on blood culture day
Global Schoenfeld Test p: 0.1276
Schoenfeld Individual Test p: 0.3036
Beta(t) for plt_lower_100
Time
Plt <10^5 /L
Global Schoenfeld Test p: 0.1276
Schoenfeld Individual Test p: 0.0371
Beta(t) for aki
Time
Acute Kidney Injury

S-5: Subclassifications of BSI source by period 1 and 2

**Characteristic**	**Period 1**, N = 84[1]	**Period 2**, N = 73[1]	***p*-value**[2]
Bacteremia Source			0.3
Primary	23 (27%)	22 (30%)	
Complicated UTI	9 (11%)	4 (5.5%)	
Pneumonia	21 (25%)	18 (25%)	
Hepatobiliary	7 (8.3%)	2 (2.7%)	
Catheter Inf	19 (23%)	25 (34%)	
Complicated SSTI	5 (6.0%)	2 (2.7%)	

[1]n (%)

[2]Fisher's exact test

S-6 Multiple Stratification and Analysis of Outcome by Period and Resistance Type

	**Period 1**, N = 84				**Period 2**, N = 73			
**Characteristic**	**DTR**, N = 9[1]	**MDR**, N = 13[1]	**None**, N = 62[1]	**p-value**[2]	**DTR**, N = 28[1]	**MDR**, N = 4[1]	**None**, N = 41[1]	***p*-value**[2]
**Outcome**				0.2				0.3
Recover	4 (8.5%)	5 (11%)	38 (81%)		18 (45%)	1 (2.5%)	21 (53%)	
Death	5 (14%)	8 (22%)	24 (65%)		10 (30%)	3 (9.1%)	20 (61%)	
**Ventilation support**				0.070				0.9
Nasal	1 (10%)	0 (0%)	9 (90%)		0 (0%)	0 (0%)	1 (100%)	
NIMV	4 (31%)	2 (15%)	7 (54%)		3 (50%)	0 (0%)	3 (50%)	
MV	4 (6.6%)	11 (18%)	46 (75%)		25 (38%)	4 (6.1%)	37 (56%)	
Bacteremia Source				0.2				0.3
Primary	1 (4.3%)	3 (13%)	19 (83%)		6 (27%)	2 (9.1%)	14 (64%)	
Complicated UTI	0 (0%)	4 (44%)	5 (56%)		3 (75%)	0 (0%)	1 (25%)	
Pneumonia	3 (14%)	1 (4.8%)	17 (81%)		9 (50%)	2 (11%)	7 (39%)	
Hepatobiliary	1 (14%)	2 (29%)	4 (57%)		1 (50%)	0 (0%)	1 (50%)	
Catheter Inf	3 (16%)	3 (16%)	13 (68%)		9 (36%)	0 (0%)	16 (64%)	
Complicated SSTI	1 (20%)	0 (0%)	4 (80%)		0 (0%)	0 (0%)	2 (100%)	

	Period 1, N = 84				Period 2, N = 73			
**Characteristic**	**DTR**, N = 9[1]	**MDR**, N = 13[1]	**None**, N = 62[1]	**p-value**[2]	**DTR**, N = 28[1]	**MDR**, N = 4[1]	**None**, N = 41[1]	***p*-value**[2]

[1]n (%)

[2]Fisher's exact test
